# Senescent cells at the crossroads of aging, disease, and tissue homeostasis

**DOI:** 10.1111/acel.13988

**Published:** 2023-09-20

**Authors:** Chisaka Kuehnemann, Christopher D. Wiley

**Affiliations:** ^1^ Jean Mayer USDA Human Nutrition Research Center on Aging at Tufts University Boston Massachusetts USA

**Keywords:** cellular senescence, disease drivers of aging, homeostasis, progeria, senolytics

## Abstract

Originally identified as an outcome of continuous culture of primary cells, cellular senescence has moved beyond the culture dish and is now a bona fide driver of aging and disease in animal models, and growing links to human disease. This cellular stress response consists of a stable proliferative arrest coupled to multiple phenotypic changes. Perhaps the most important of these is the senescence‐associated secretory phenotype, or senescence‐associated secretory phenotype —a complex and variable collection of secreted molecules release by senescent cells with a number of potent biological activities. Senescent cells appear in multiple age‐associated conditions in humans and mice, and interventions that eliminate these cells can prevent or even reverse multiple diseases in mouse models. Here, we review salient aspects of senescent cells in the context of human disease and homeostasis. Senescent cells increase in abundance during several diseases that associated with premature aging. Conversely, senescent cells have a key role in beneficial processes such as development and wound healing, and thus can help maintain tissue homeostasis. Finally, we speculate on mechanisms by which deleterious aspects of senescent cells might be targeted while retaining homeostatic aspects in order to improve age‐related outcomes.

## Graphical abstract

Originally identified as a phenotype adopted by cultured cells during replicative exhaustion, cellular senescence has expanded beyond the culture dish and is now a recognized driver of aging. However, the physiological role of senescence is complex, promoting tissue homeostasis or driving pathology depending on context. Kuehnemann and Wiley review the physiological role of senescent cells in the context of disease drivers of aging and tissue homeostasis, with an eye toward targeting the complexity of senescence to distinguish between these processes.

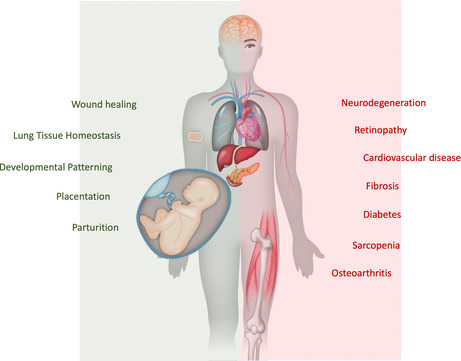

**Abbreviations**
ART: antiretroviral therapy
ATV: atazanavir
CGAS: cyclic GMP–AMP synthase
FTC: emtricitabine
HDAC: histone deacetylase
HGPS: Hutchinson‐Guildford Progeria Syndrome
HIV: human immunodeficiency virus
LMNA: lamin A
LMNB1: lamin B1
mtDNA: mitochondrial deoxyribonucleic acid
NRTI: nucleotide reverse transcriptase inhibitor
PI: protease inhibitor
PrEP: pre‐exposure prophylaxis
SASP: senescence‐associated secretory phenotype
STING: stimulator of interferon genes
TDF: tenofovir disoproxil fumarate

## 1 FROM DISCOVERY IN CULTURE TO DRIVERS OF DISEASES

Prior to the discovery of senescence in the 1960s, it was commonly asserted that cells had essentially unlimited divisional potential in culture, as proposed by Alex Carrel decades earlier (Carrel, 1912). The finding that normal fibroblasts eventually stop dividing and assume a state of stable proliferative arrest challenged this view, but also suggested a possible rationale for tumorigenesis, as unlimited life spans in culture were more often found to be associated with oncogenic transformation (Hayflick & Moorhead, 1961). In the intervening years, senescence was indeed confirmed as a tumor suppressive process (Sager, 1991). The cellular phenotypes that accompany senescence have likewise expanded in recent years, adding substantial complexity to the study of senescence, but also multiple vectors by which senescent cells may have effects that extend far beyond simple arrest.

### 1.1 Complexity of senescence phenotypes

Not every cell that no longer divides is senescent, or every neuron in the brain or podocyte in the kidney would be senescent (Gorgoulis et al., 2019). Rather, the proliferative arrest is only one of a complex spectrum of phenotypes. Perhaps the most important of these phenotypes is the secretion of a multitude of biologically active molecules that can have important effects on the surrounding cells and tissue. This senescence‐associated secretory phenotype (SASP) consists of a combination of inflammatory cytokines, chemokines, growth factors, proteases, oxylipins, and other signaling molecules. The SASP can therefore explain how relatively few senescent cells might drive multiple conditions associated with aging. Much of the SASP is proinflammatory, and thus senescent cells are a prime candidate for driving age‐associated inflammation, or “inflammaging” (Franceschi & Campisi, 2014). Beyond inflammation, the SASP has additional properties including promoting hemostasis, fibrosis, tumor growth and invasion, and even paracrine induction of senescence in other cells (Acosta et al., 2013; Coppe et al., 2008; Schafer et al., 2017; Wiley, Brumwell, et al., 2019; Wiley, Liu, et al., 2019). For this reason, the SASP is an important target for the prevention of multiple age‐related conditions.

Inducers of senescence are many and varied, but typically result from some form of cellular stress that initiates the early events in the senescence program. These include—but are not limited to—genotoxic DNA damage, telomere uncapping, aneuploidy, mitochondrial dysfunction, oncogene activation, and HDAC inhibition (Andriani et al., 2016; Bodnar et al., 1998; Ogryzko et al., 1996; Serrano et al., 1997; Wiley et al., 2016). The SASP can vary with time, cell type, and inducer—adding additional complexity and contextual nuance to an already complex process (Basisty et al., 2020; Coppe et al., 2008, 2010; Hernandez‐Segura et al., 2017; Sturmlechner et al., 2021; Wiley et al., 2016). While still somewhat unclear, it is likely that this variation in SASP may also contextually extend to diseases driven by senescent cells.

Senescent cells also display disruptions in organelle homeostasis, including mitochondrial and lysosomal dysfunction (Correia‐Melo et al., 2016; Johmura et al., 2021), autophagic dysregulation (Kang et al., 2015; Young et al., 2009), as well as nuclear lamina and chromatin modifications (Dou et al., 2015; Freund et al., 2012; Narita et al., 2003). These disruptions can also drive cell cycle arrest and elements of the SASP. For example, selective autophagy of the transcription factor GATA4 is lost during senescence, which in turn drives multiple senescence phenotypes (Kang et al., 2015). Conversely, autophagic degradation of lamin B1 drives laminopathy and reinforces senescence (Dou et al., 2015). Importantly, disruption of either nuclear or mitochondrial integrity results in release of DNA into the cytosol. These DNAs activate the CGAS‐STING signaling pathway, which detects cytosolic DNA and leads to an inflammatory interferon response similar to that observed in viral infection. Three major sources of cytosolic DNA have been identified in the context of senescence. Loss of nuclear lamin B1 (LMNB1), especially following genotoxic stress, results in release of small amounts of cytosolic chromatin (Dou et al., 2017). Additionally, over time senescent cells lose repression of LINE‐1 elements, the reverse‐transcribed complimentary DNA produced during this process can then accumulate in the cytosol (De Cecco et al., 2019). Finally, mitochondrial DNA (mtDNA) can be released by dysfunctional mitochondrial in senescent cells (Iske et al., 2020). Between these mechanisms, there are multiple pathways by which CGAS can be activated to promote the SASP and its downstream effects.

Since senescence phenotypes are complex, variation in these phenotypes can occur, leading to questions regarding not only the detection of senescent cells, but also in some cases how we might define senescence. Indeed, even the most notable senescence phenotype, the proliferative arrest, has been reported to be reversible in some contexts (Fleury et al., 2019; Galanos et al., 2016; Kuehnemann et al., 2023; Milanovic et al., 2018; Saleh et al., 2019). In these cases, the reversal of proliferative arrest is often coupled to the loss of the stressor that drove the senescent phenotype, as seen in the case of drug removal (Fleury et al., 2019; Kuehnemann et al., 2023), or in cancer following mutation/reprogramming of a tumor suppressor pathway (Galanos et al., 2016; Milanovic et al., 2018). Conceptually, the idea of reversibility of senescence is still somewhat controversial, as the irreversibility of senescence is often held as a defining feature. Similarly, can an inherently postmitotic cell type such as a neuron undergo senescence if there is no proliferation to arrest? For example, neurons and cardiomyocytes have been shown to express markers of senescence, suggesting that cell cycle competence may not be required for at least some aspects of the senescent phenotype (Anderson et al., 2019; Herdy et al., 2022; Jurk et al., 2012). Variation in senescence phenotypes and attempts to build consensus for study in the field were the subject of a recent perspective from several members of the International Cellular Senescence Association (Gorgoulis et al., 2019).

### 1.2 Relationship to aging

**FIGURE 1:**
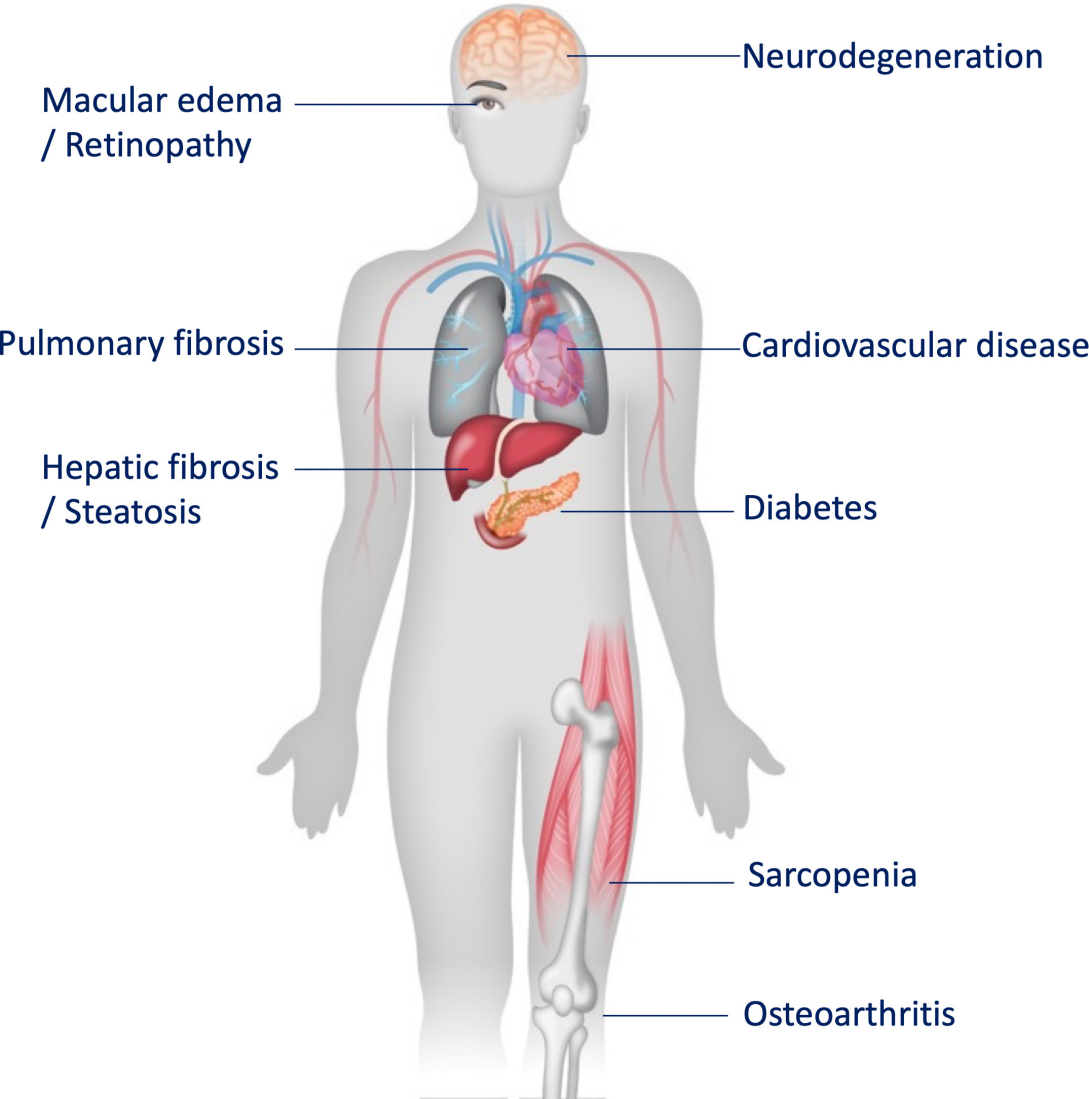
Pathological consequence of senescence. Senescent cells promote degeneration conditions in multiple organ systems. In the eye they can promote macular edema, retinopathy, and cataracts. In the brain senescent cells can drive neurodegeneration and outcomes that resemble Parkinson's or Alzheimer's disease. In the lung and liver, senescent cells can promote fibrosis. Both cardiac and endothelial senescent cells can promote cardiovascular disease. Senescent pancreatic beta cells drive diabetes, and in the musculoskeletal system senescent cells drive both sarcopenia and osteoarthritis. These represent a subset of conditions shown to be targetable by senotherapeutic strategies in animal models.

Once only suspected to be linked to aging, a large body of the literature demonstrating a causal role between senescence and age‐related diseases has grown out of studies using transgenic mouse models to either track the accumulation of senescent cells, eliminate them, or both. Initially, most of these mice expressed a variety of transgenes from the *p16* ^*INK4a*^ promoter (Baker et al., 2011; Burd et al., 2013; Demaria et al., 2014), but have recently expanded to include transgenes targeted to *p21* ^*WAF1/CIP1*^ (Wang et al., 2021). The models that selectively eliminate these cells have proven especially important, demonstrating that p16‐ and p21‐positive cells limit both life span and health span (Baker et al., 2011; Wang et al., 2021). In the intervening years, these models and interventions that selectively kill senescent cells (commonly called senolytics) have been efficacious in preventing or treating a number of age‐related conditions including osteopenia, atherosclerosis and cardiovascular dysfunction, alopecia, glomerulosclerosis, cataracts and other eye diseases, hepatic fibrosis and steatosis, osteoarthritis, immune suppression, pulmonary fibrosis, coagulation, arthritis, and diabetes (Aguayo‐Mazzucato et al., 2019; Amor et al., 2020; Baar et al., 2017; Baker et al., 2016; Chang et al., 2016; Childs et al., 2016; Farr et al., 2017; Jeon et al., 2017; Ogrodnik et al., 2017; Palmer et al., 2019; Schafer et al., 2017; Thompson et al., 2019; Wiley, Liu, et al., 2019). Thus, senescent cells influence both aging and development of chronic degenerative diseases in mice (Figure 1).

Discovery of the first biomarker of senescence, senescence‐associated beta‐galactosidase, allowed verification that senescence occurs in vivo (Dimri et al., 1995). With this came the observation that senescent cells increase in humans during aging. While most direct evidence for senescent cells driving aging and degenerative disease comes from mice, a number of studies have established associations between senescence and human aging and disease. For example, one major contribution of a recent mass spectrometry‐based SASP atlas is a notable overlap between multiple core SASP factors and blood markers of human aging from the Baltimore Longitudinal Study of Aging (Basisty et al., 2020). Other sets of SASP factors have similarly been linked to age and frailty (Schafer et al., 2020) and loss of physiological function (Fielding et al., 2022). Conversely, human centenarian and calorie restriction studies are linked to lower levels of some SASP markers (Fontana et al., 2018; Sebastiani et al., 2021). Combined with mouse and cell culture data, these studies suggest that senescent cells are likely to drive age‐related diseases and limit life span in humans, but due to the complexity of the SASP (>1000 factors identified across different inducers, tissue, and cell types), it remains difficult to definitively state that senescent cells are the driving factor based solely on secreted biomarkers.

Despite these observations linking disease outcomes, perhaps the best evidence for senescent cells driving age‐associated conditions comes from diseases that drive premature aging. We review this evidence below.

## 2 SENESCENT CELLS IN DISEASES OF PREMATURE AGING

**FIGURE 2:**
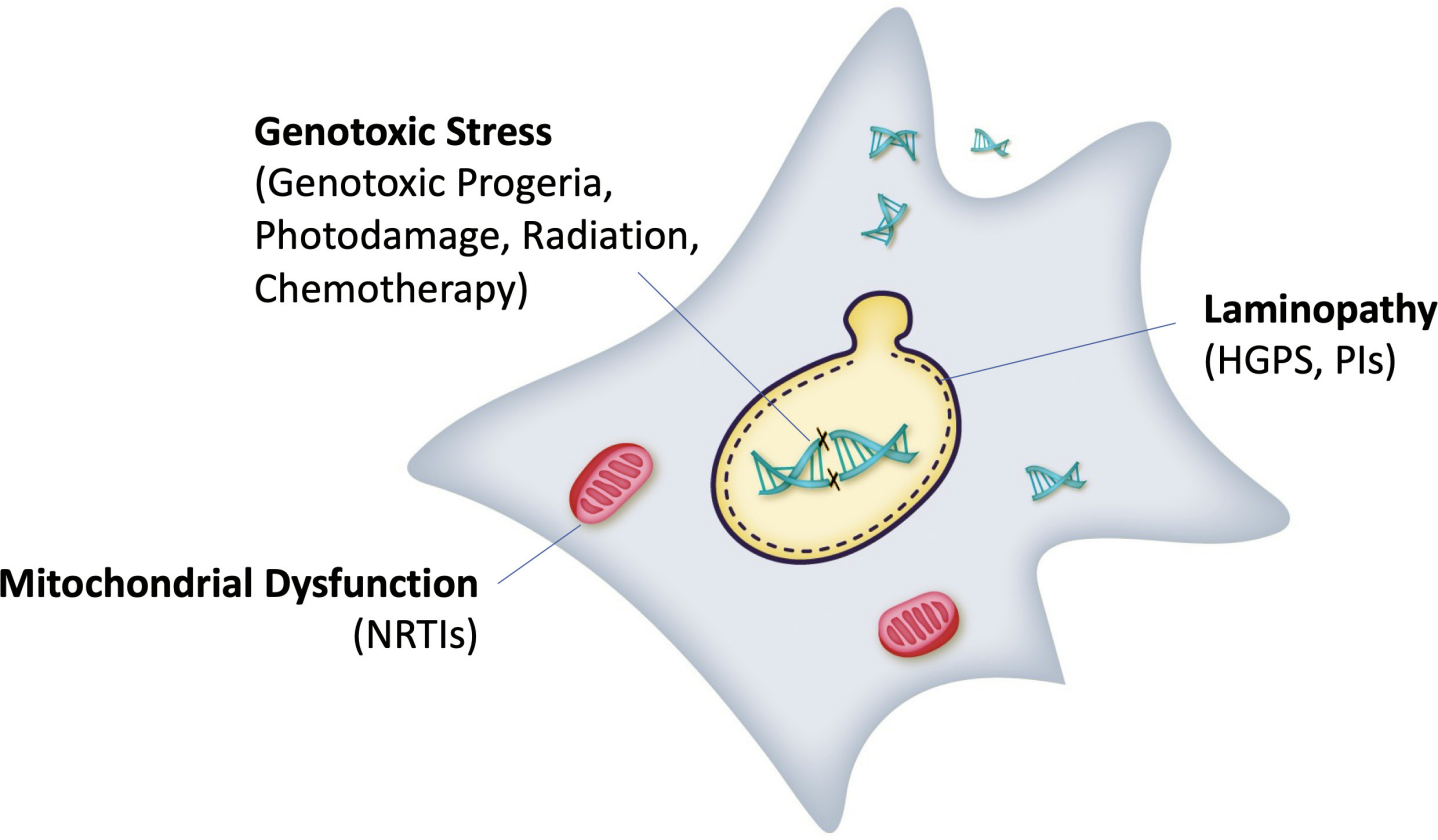
Disease drivers of senescence. Multiple chronic diseases and their treatments result in both aging and cellular senescence. Genotoxic progerias, photodamage, and cancer treatments such as radiation and chemotherapy drive senescence through strand breaks and other forms of DNA damage. Nucleotide reverse transcriptase inhibitors (NRTIs) used in HIV therapy can deplete or cause mutations in mitochondrial DNA. Finally, progeroid laminopathies such as Hutchinson‐Guilford Progeria Syndrome (HGPS) and protease inhibitors (PIs) used for HIV therapy can drive nuclear lamina disruption. Each of these in turn can drive senescence and potentially promote age‐related pathologies.

The role of senescence in aging is revealed at least in part by the finding that senescence is often associated with conditions that drive or accelerate the development of age‐associated diseases. These include genetic disorders that result in premature aging (progerias), cancer and HIV therapies, as well as complications of diabetes and metabolic syndrome (Figure 2).

### 2.1 Progeria

Several genetic syndromes are associated with features of premature aging, also known as progerias. While the features associated with premature aging vary with the effected gene, there are broadly two classes of progeroid drivers: laminopathies, which result from disruptions in the nuclear lamina, and genotoxic progerias, which result from loss of DNA repair. Perhaps the best‐studied progeria is a laminopathy known as Hutchinson–Guilford Progeria Syndrome (HGPS), a condition with multiple features of premature aging. Children with HGPS present with alopecia, micrognathia, growth restriction, skin wrinkling, loss of eyesight, and cardiovascular disease, the last of which is a frequent cause of death (Gordon et al., 1993). Patients with HGPS rarely live past their late teens. While wild‐type lamin A (LMNA) can be cleaved by the intracellular protease zmpste24 into a functional mature form; in HGPS a mutation in lamin A results in a cryptic splice variant that cannot be cleaved, resulting in accumulation of the premature form, ultimately disruption the nuclear lamina. Additionally, another form of human laminopathy, restrictive dermopathy, results from mutations in *ZMPTSE24*, the enzyme that cleaves prelamin A into mature lamin A—resulting in accumulation of prelamin A, much like in HGPS (Navarro et al., 2004). Phenotypes associated with restrictive dermopathy include birth with tight, thin skin that readily cracks, micrognathia, sparse or absent eyelashes, and lipoatrophy, typically resulting in perinatal lethality. Mouse knockouts of *Zmpste24* show features of premature aging (Bergo et al., 2002; Pendas et al., 2002). In both the human disease and the mouse models, premature aging is linked to either accumulation of senescent cells or rapid senescence in culture (Huang et al., 2008; Varela et al., 2005). Additionally, it is possible that the disruption of nuclear architecture might result in cytosolic DNA accumulation, as observed during lamin B1 loss in senescent cells, raising the possibility that some of the phenotypes observed in HGPS result from a SASP‐like phenotype induced by cytosolic DNA‐driven CGAS‐STING signaling (Gonzalo & Coll‐Bonfill, 2019), potentially in the absence of senescence. Together, these data indicate that maintenance of nuclear lamina homeostasis is essential for preventing age‐related phenotypes associated with senescence and the SASP.

Additionally, genotoxic progerias, such as those seen in Werner's Syndrome, Cockayne Syndrome, and trichothiodystrophy, similarly result in segmental phenotypic features of aging (Burtner & Kennedy, 2010). Since genotoxic stress is a key driver of senescence, it is not surprising that many of these diseases have been linked to the accumulation of senescent cells. Cells isolated from patients with Werner's syndrome show increased senescence markers, and mouse models of the disease have increased DNA damage, *p16* ^*INK4a*^ expression, and senescence‐associated β‐galactosidase (Lu et al., 2014; Norwood et al., 1979). Cockayne syndrome patients present with growth failure, microcephaly, neurodegeneration, cataracts, and are sensitive to UV‐induced DNA damage (Aamann et al., 2013; Pasquier et al., 2006), and skin cells from individuals with Cockayne syndrome show elevated markers of senescence (Cordisco et al., 2019). Similarly, trichothiodystrophy is a disorder resulting in brittle hair and nails, premature birth and growth retardation, and photosensitivity (Tay, 1971). A mouse model of this disease prematurely accumulates senescent cells, and elimination of senescent cells improves phenotypes in this model (Baar et al., 2017). As such, there are multiple animal models of genotoxic progerias that also result in accumulation of senescent cells (Yousefzadeh et al., 2019). Several additional DNA repair disorders have been shown to play a role in cardiovascular diseases, neurodegeneration and cancer (Keijzers et al., 2017; Petr et al., 2020). Thus, multiple genetic drivers of premature aging are also drivers of senescence and the SASP, suggesting that senescence may be a key age‐promoting downstream effector of these drivers. Importantly, not all DNA repair deficiencies drive premature aging or senescence, suggesting that the origin of the genotoxic lesion may be important in determining whether cancer or senescence and other aging phenotypes manifest.

### 2.2 Cancer and its therapies

Since senescence results in stable arrest of proliferation, senescence can be considered a tumor suppressive process. Thus, senescence may be considered a desirable outcome for cancer therapies in the short term (Campisi, 2003a; Lecot et al., 2016). In agreement with this, many cancer therapies, including both radiation and chemotherapies, result in the senescence of cancer cells (Schmitt et al., 2022). This is often due to genotoxic damage and/or aneuploidy, but can potentially originate from other drivers. Such therapies can also drive either transient or chronic conditions common in aging, including loss of hair and muscle mass, immune suppression, thrombotic events, cardiovascular disease, fibrotic disease, and—ironically—cancer relapse and invasion, many of which can be prevented by eliminating senescent cells (Demaria et al., 2017; Limbad et al., 2022; Schafer et al., 2017; Wiley, Liu, et al., 2019). Elimination of senescent cells following treatment with chemotherapeutics can prevent many of these conditions, offering a potential one‐two punch in which cancer can be targeted by therapies that drive senescence, but then allow removal of senescent cells by senolysis to improve outcomes. This approach has additionally been used to fight cancer in preclinical models by first therapeutically inducing senescence of tumor cells, then eliminating these cells via senolytic therapy to drive synthetic lethality (Fleury et al., 2019; Wang et al., 2019). These combinations may improve future cancer therapies by exploiting the tumor suppressive properties of senescent cells while preventing their more deleterious effects by clearing them before they can cause lasting harm.

### 2.3 HIV and its therapies

While not a progeroid disorder in and of itself, long‐term infection with HIV is linked to a number of phenotypes that collectively resemble aging, including lipodystrophy, osteoporosis, type 2 diabetes, cardiovascular disease, neurodegenerative disease, and cancer (Smith et al., 2012). HIV‐infected patients have a an 8‐year shorter life expectancy relative to those without infection, even following early initiation of antiretroviral therapy (ART) (Marcus et al., 2016). Chronic immune activation and toxic side effects of ART medications have been implicated as contributing factors to the observed aging pathologies (Pathai et al., 2014), and these increased immune responses can contribute to the pathogenesis of age‐related diseases. While these and many other factors (e.g., psychosocial stress) could contribute to both premature aging and accumulation of senescent cells during HIV infection, we will focus on use of ART as an inducer of senescence, as these have been clearly demonstrated to drive senescence in cultured cells. While long‐term antiretroviral use is associated with serious side effects that resemble premature aging (Lohse et al., 2007), the individual influence of ART on disease susceptibility is difficult to define, as these drugs are essential for patient survival in the long term, and uninfected individuals did not take ART drugs prior to the introduction of pre‐exposure prophylaxis (PrEP) in 2012. However, data from mouse and cell culture models show that senescent cells increase following treatment with certain HIV therapies, suggesting that they may promote age‐related phenotypes and pathologies in patients. ART regimens generally consist of a combination of two nucleoside reverse transcriptase inhibitors (NRTIs) with a third inhibitor from one of three antiretroviral drug classes including integrase strand transfer inhibitors, nonnucleoside reverse transcriptase inhibitors (NNRTIs), or protease inhibitors (PIs) (Shafer & Vuitton, 1999), and accumulating evidence from cell culture and mouse models of ART treatment implicates both NRTIs and PIs in the pro‐aging side effects of ART, as well as development of senescent cells.

#### 2.3.1 NRTIs and mitochondrial dysfunction

Patients that receive nucleotide reverse transcriptase inhibitors (NRTIs) often show signs of premature aging, including lipodystrophy, osteoporosis, cardiomyopathy, and diabetes (Torres & Lewis, 2014). Some NRTIs can inhibit the mammalian mitochondrial DNA polymerase gamma (POLG) responsible for mitochondrial DNA (mtDNA) synthesis and thus lead to depletion of mtDNA, resulting in mitochondrial dysfunction (Apostolova et al., 2011), and POLG mutant mice that accumulate mtDNA mutations or lose mtDNA at an accelerated rate also age prematurely (Kujoth et al., 2005; Singh et al., 2018; Trifunovic et al., 2004). Inducers of mitochondrial dysfunction can cause or accelerate cellular senescence, and this mitochondrial dysfunction‐associated senescence (MiDAS) has a distinct secretory phenotype that promotes disorders such as lipoatrophy (Wiley et al., 2016). NRTIs induce a similar senescent phenotype to other inducers of mitochondrial dysfunction. For example, zidovudine (AZT) and stavudine (d4T), which were among the first drugs to treat HIV infection, are strong inducers of mitochondrial dysfunction. Fat biopsies from patients with NRTI‐induced lipodystrophy accumulate senescent cells (Caron et al., 2008), providing a link between senescence and the toxic effects of these NTRI therapies. The NRTIs emtricitabine (FTC) and tenofovir disoproxil fumarate (TDF) are prescribed together in pre‐exposure prophylaxis (PrEP) to reduce the risk of contracting HIV in HIV‐negative individuals. Cardiac fibroblasts and endothelial cells treated with FTC/TDF in combination results in mitochondrial dysfunction and cellular senescence (Chen et al., 2019; Cohen et al., 2018; Nacarelli et al., 2016) suggesting the possibility that at least some NRTIs may accelerate cardiovascular disease by inducing a state of mitochondrial dysfunction in these cell types. Together, these studies identify a potential role for senescent cells in mediating toxic effects of NRTIs, though in vivo evidence for senescent cells as causal agents of these phenotypes is missing, presenting an area for future study.

#### 2.3.2 Protease inhibitor‐induced laminopathy

Additionally, mouse and cell culture models of HIV protease inhibitor (PI) treatment suggests that this drug class can activate pro‐aging biological processes including cellular senescence (Auclair et al., 2014; Caron et al., 2003, 2007; Kuehnemann et al., 2023). PIs lower HIV viral load by targeting the protease responsible for proteolytic cleavage of viral proteins into their mature form, thereby blocking proper viral packaging (Nolan, 2003). Unfortunately, PI treatment can also impair maturation of LMNA by inhibiting the activity of ZMPSTE24, thus inducing laminopathies in a manner similar to progeroid disorders (Coffinier et al., 2007, 2008; Young et al., 2005). When healthy fibroblasts are treated with the HIV PIs indinavir or nelfinavir, or a combination of atazanavir and ritonavir, they accumulate prelamin A and senescence phenotypes (Caron et al., 2007; Kuehnemann et al., 2023), potentially linking these therapies to senescent cells associated with progeroid disorders.

Importantly, treatment with atazanavir and ritonavir not only induces markers of senescence in cultured cells and animal models, but also removal of these drugs results in loss of senescence phenotypes in culture and lowers senescent cell burden in mice (Kuehnemann et al., 2023). Cessation of treatment in animals also improves functional outcomes, an important finding for patients, who may experience improvement by switching therapies. These reversal data complement findings that correction of the LMNA mutation by base editing in 20%–60% of cells in HGPS model mice improves pathology and partially rescues life span effects (Koblan et al., 2021), suggesting that these effects are not permanent and can be improved if the source of laminopathy is removed. Overall, senescent cells are consistently present at sites of many age‐related pathologies also associated with PI‐treatment. It is therefore worth further consideration that senescent cells might underlie some of the toxic effects of HIV PIs.

### 2.4 Diabetes and metabolic disease

A growing body of literature has revealed links between senescent cells and diabetes. These processes appear to be interconnected, as senescent cells can promote diabetes and metabolic disease, which in turn appears to drive senescence. Initially, senescence was linked to diabetes in the pancreas, where *p16* ^*INK4a*^ expression associated with increased insulin secretion (Helman et al., 2016). However, as senescent beta cells increase insulin secretion, they also promote immune recruitment and destruction (Aguayo‐Mazzucato et al., 2019; Thompson et al., 2019). Additionally, peripheral senescent cells, such as in adipose tissue of mice with diet‐induced obesity, can promote insulin resistance outside the pancreas (Palmer et al., 2019). Finally, diabetes can result in senescent cells in nonmetabolic tissues. For example, diabetes can drive senescent renal cells, which then promote diabetic kidney disease (Kim et al., 2019; Verzola et al., 2008). Similarly, diabetes can drive senescence in the eye, which in turn drives conditions such as diabetic retinopathy (Crespo‐Garcia et al., 2021). Thus, senescence is both a driver and a consequence of diabetes—and a potential vector by which diabetes accelerates age‐related degenerative pathologies.

These studies come with some limitations. For example, elimination of senescent cells in aged mice had no effect on glucose or insulin tolerance (Baker et al., 2016), and diabetes is not a common consequence of progerias, chemotherapies, or most HIV treatments in use today—though earlier HIV therapies were associated with diabetes (Brambilla et al., 2003; Iwata & Ogawa, 2017; Kalra et al., 2011). It is currently unclear why this is the case. One possibility is that only metabolic disease might drive pancreatic beta cell senescence, but this does not explain why diet‐induced senescent adipose cells drive insulin resistance (Palmer et al., 2019), while other drivers of senescence in the same tissue have not been reported to do so. Alternatively, since senescence and the SASP can vary by cell type and inducer (Basisty et al., 2020; Coppe et al., 2008; Wiley et al., 2016), it may be the case that induction of senescence by macronutrient stressors drives a specific secretome that promotes insulin resistance. These questions represent fertile ground for future studies.

Overall, it appears that senescent cells and their related pathways are associated with multiple conditions that drive premature degenerative pathologies that collectively resemble aging. Thus, senescent cells and their drivers may be key determinants of the rate in which we age and develop degenerative disease. A key question arises from these observations: if senescent cells are deleterious for so many conditions, why did this process evolve and how can it be beneficial? We next review the beneficial aspects of senescence and how they promote tissue homeostasis.

## 3 SENESCENT CELLS PROMOTE TISSUE HOMEOSTASIS

**FIGURE 3:**
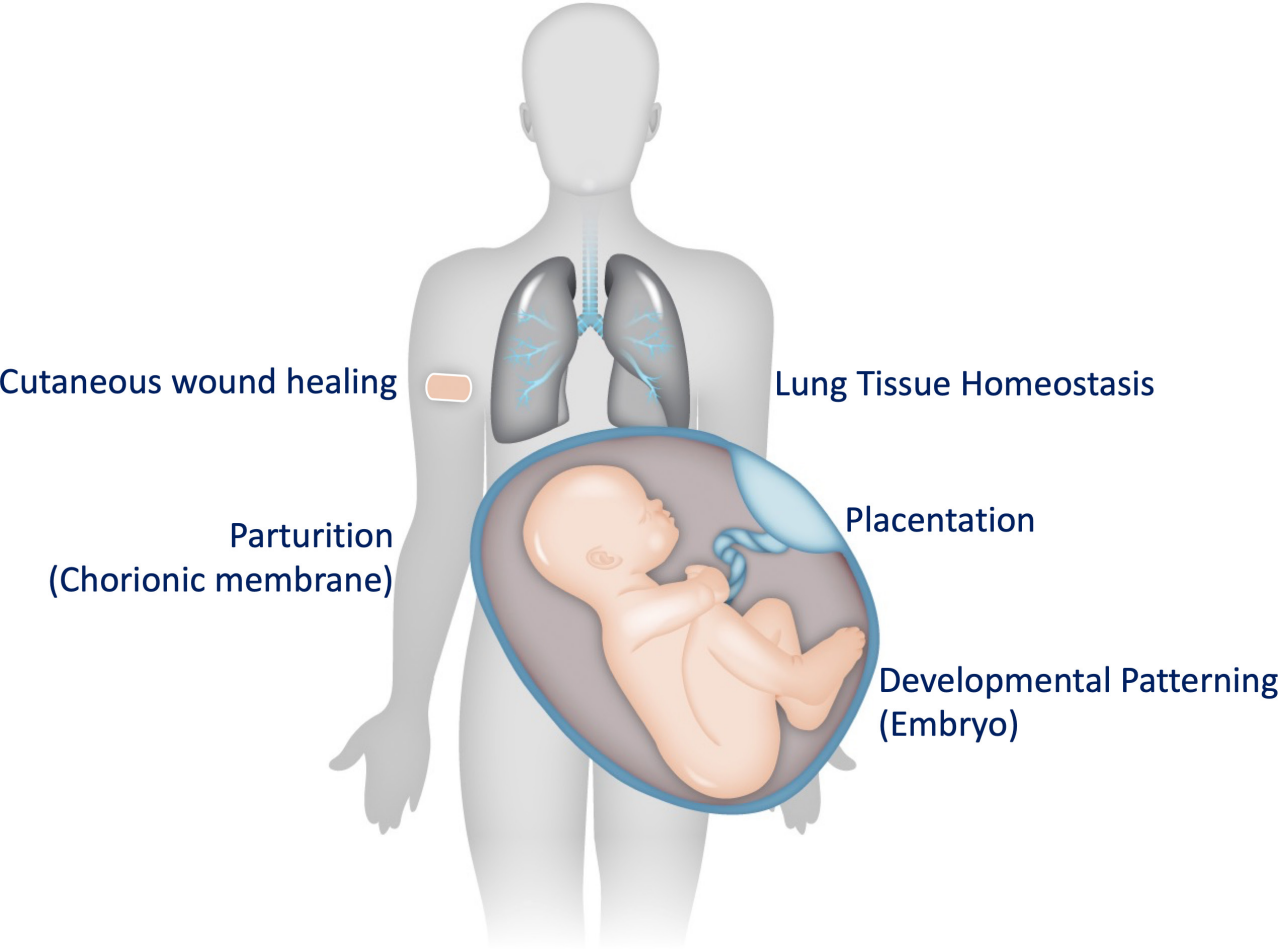
Homeostatic mechanisms of senescence. Senescent cells are required for multiple homeostatic processes. They appear at sites of cutaneous wounds to promote healing, and can reside in lung tissue until required to help regeneration. During development senescent cells appear in the embryo to drive pattern formation and promote placentation. Finally in the chorionic membrane, senescent cells can send signals the promote parturition.

While senescence and the SASP can promote many deleterious conditions, a growing body of evidence reveals a role for senescence in many homeostatic physiological processes (Figure 3).

### 3.1 Tumor suppression

As mentioned above, the role of senescence in tumor suppression is clear: Stable cell cycle arrest associated with senescence prevents proliferation of malignant and premalignant cells. Indeed, loss of members of the arrest signaling pathways that promote senescence, including p16^INK4a^, p15^INK4b^, p14/p19^ARF^, p53, or Rb all result in increased rates of cancer (Donehower et al., 1992; Jacks et al., 1992; Krimpenfort et al., 2007; Pomerantz et al., 1998; Sharpless et al., 2001). Mutations in senescence‐promoting pathways results in human cancer susceptibilities such as Li‐Fraumeni Syndrome and retinoblastoma (Lee et al., 1987; Malkin, 2011), and other members of these pathways are frequently mutated in human cancers (Vogelstein et al., 2013). Thus, senescence is an essential barrier to the progression of cancer. The role of senescence in cancer progression has been reviewed extensively elsewhere (Campisi, 2003a, 2013; Schmitt et al., 2022; Wyld et al., 2020).

Importantly, it is unlikely that tumor suppression is the singular reason for the evolutionary origin of senescent cells, as apoptosis and other cell death pathways appear in virtually every metazoan and even some nonanimals (Ameisen, 2002)—and apoptosis is also tumor suppressive (Delbridge et al., 2012). The discovery of additional roles for senescent cells beyond tumor suppression supports this notion. These include:

### 3.2 Development

Senescent cells are essential for multiple aspects of mammalian development. In extraembryonic tissues, senescent cells appear during placental development, where they play an important role in uterine invasion and normal development (Velarde & Menon, 2016). For example, syncytiotrophoblasts—a key cell type at the maternal/fetal interface—are multinucleate cells that exhibit multiple markers of senescence, and these markers are lower or even absent in cases of human intrauterine growth restriction (Gal et al., 2019). Senescent cells also appear to be active participants in parturition, as they appear in the chorionic membrane immediately prior to live birth and trigger parturition (Behnia et al., 2015).

In the developing mouse embryo, senescent cells appear in a TGF‐B‐ and PI3K/FOXO‐dependent manner, where they are eventually cleared by macrophages (Munoz‐Espin et al., 2013). These developmental senescent cells assist in structure development in the apical ectodermal ridge (Storer et al., 2013) and in the mesonephros and endolymphatic sac (Munoz‐Espin et al., 2013). Senescence is also conserved in amphibian development, including *Xenopus* and the axolotl (Davaapil et al., 2017). The axolotl is uniquely in a paedomorphic state and possesses strong regenerative capabilities, including the ability to regrow limbs (Monaghan et al., 2014). Senescent cells appear at limb buds in frogs, and during limb regrowth in the axolotl in a TGF‐beta‐dependent manner, as in murine limb development (Munoz‐Espin et al., 2013; Storer et al., 2013), and are rapidly removed by immune cells (Davaapil et al., 2017; Yun et al., 2015). Inhibition of TGF‐beta both prevents the accumulation of senescent cells and disrupts developmental patterning in these models.

### 3.3 Cutaneous wound healing

Senescent cells also appear during wound healing in adult mice (Demaria et al., 2014; Jun & Lau, 2010) and humans (Chia et al., 2021). In mice, elimination of senescent cells slows wound closure and results in increased scarring after wound closure (Demaria et al., 2014). Notably, these effects of senescent cells are acute in nature, as age‐related chronic accumulation of senescent cells can slow wound healing (Thanapaul et al., 2022; Velarde et al., 2015). Thus, senescence is beneficial when acute in wound healing, but detrimental when chronic, highlighting the importance of timing as a contextual variable when considering the role of senescent cells in disease.

### 3.4 Maintenance of tissue homeostasis

Senescence can also maintain tissue homeostasis in response injury or other challenges. For example, senescence in hepatic stellate cells limits the development of fibrosis in response to injury by carbon tetrachloride (Krizhanovsky et al., 2008) though elimination of these cells, once established, also antagonizes fibrosis in this model (Amor et al., 2020)—highlighting the complex relationship between these processes. Furthermore, senescent fibroblasts appear in the basement membrane adjacent to the epithelial stem cell niche in the lung during postnatal tissue maturation. These lay dormant within the airway stem cell niche, but upon injury to the airway epithelium increase their secretory phenotype to promote epithelial regeneration and restore barrier integrity (Reyes et al., 2022). Conversely, induction of senescence in the lung can drive pulmonary fibrosis, and elimination of senescent cells by senolytics or blockade of NOX4 can reverse established and progressive fibrosis (Hecker et al., 2014; Pan et al., 2017). Thus, depending on context, senescence can be either a homeostatic or degenerative mechanism in the same tissue—an important area for further study.

Together, these studies highlight the importance of senescent cells as a homeostatic process and argue for contextual benefits to their accumulation. These benefits of senescence are likely to favor survival early in life, and development of pathology later in life. As such, senescent cells may a consequence of antagonistic pleiotropy—an evolutionary hypothesis that posits that traits that benefit an organism early in life might become detrimental later in life when selective pressure for the trait declines (Campisi, 2003b; Williams, 1957). This idea has been contested, primarily on the grounds that beneficial aspects of senescence are not necessarily linked to young ages (Giaimo & di d'Adda Fagagna, 2012). However, this was prior to demonstration of the role of senescence in development and live birth, which are early life processes, and more recent evidence for chronic senescent cells delaying wound healing (Thanapaul et al., 2022). Thus, the balance of evidence currently favors senescence as an antagonistically pleiotropic process.

## 4 THE FUTURE OF SENESCENCE THERAPIES, CAN WE TURN SLEDGEHAMMERS INTO SCALPELS?

The variation in senescent cell phenotypes creates some challenges in their elimination, as not every pathway may be shared between senescent cells (Gorgoulis et al., 2019), as is often observed during cancer (Vogelstein et al., 2013). Furthermore, beneficial aspects of senescent cells create caveats for when and how they should be eliminated, as disruption of healing, developmental, or regenerative processes in pursuit of prevention or treatment of chronic diseases is less desirable. Thus, development of next‐generation precision senolytic therapies that take advantage of potential distinctions between beneficial and detrimental senescent cells might improve safety by allowing selective clearance of those that drive the condition being treated. Since current strategies focus on targeting pathways that promote cell survival, collateral damage to other cell types, as observed in the case of thrombocytopenia with the BCL‐2 family inhibitor ABT‐263 (Schoenwaelder et al., 2011), are a current target for improvement. Indeed, a modified version of ABT‐263 that cannot be activated by platelets has removed one form of collateral damage (He et al., 2020), and others are currently in development.

One aspect of senescence that might be amenable to precision targeting is the temporal nature of acute vs. chronic senescence. Many beneficial aspects of senescence described thus far are transient in nature, and typically occur within a few days following induction/initiation of senescence (Paramos‐de‐Carvalho et al., 2021), while chronic accumulation of senescent cells has been linked to interferon activation and age‐related chronic disease (De Cecco et al., 2019). Therefore, perhaps the simplest way differentiating between beneficial and deleterious senescent cells may be by targeting effects that occur in chronic senescence and not during the more transient early response. This approach may improve outcomes in the future by improving the safety of senolytic use and more selectively only targeting those senescent cells that drive more degenerative chronic pathology.

In the absence of more targeted cell killing, interventions that selectively suppress part of the SASP might help to improve outcomes by maintaining homeostatic effects will limiting those that promote degeneration. For example, cholesterol‐lowering statins, among the most commonly prescribed drugs in the United States, can suppress proinflammatory components of the SASP (Liu et al., 2015), yet can also improve wound healing (Asai et al., 2012; Sawaya et al., 2018). These types of findings demonstrate proof‐of‐principle for selective targeting of SASP components. Importantly, due to the continued need for these therapies, SASP suppressors are likely to be less desirable for many outcomes than the more hit‐and‐run approach of senolytics, where any toxicity might be mitigated by stopping senolytic treatment after senescent cell elimination. SASP suppressors may nevertheless present an advantage in cases where senolytic therapies might not be desirable, especially in cases where multiple opposing conditions might require more precise targeting. For example, if a cancer patient has their tumor surgically removed and then is given chemotherapy, preventing senescence‐driven chemotherapy‐induced toxicity while allowing senescence‐promoted postsurgical healing in the same individual would be desirable.

Overall, identification of senescent cell types and their role in causing human disease will be invaluable in the creation of new senotherapeutics that might allow these contrasting phenotypes to be selectively targeted. This is a major potential benefit of the recently announced SenNet Consortium (SenNet, 2022), which will map senescent cells in both murine and human tissues and help to catalog the diversity, abundance, spatial localization, and secretome of senescent cells in human and murine conditions. Beyond identification of senescent cell heterogeneity, these data will also help answer questions about translatability of findings between species, identify new biomarkers of senescence, and give indications about the origins of senescent cells across the lifespan. The development of therapeutic approaches that target senescent cells is still very much in its infancy, and while animal models point toward strong potential for intervention in human disease, there is still substantial study required to confirm that these approaches are both safe and efficacious in humans.

## AUTHOR CONTRIBUTIONS

CK designed the figures for the manuscript. CK and CW conceived of the concepts and wrote the manuscript.

## CONFLICT OF INTEREST STATEMENT

CW is an inventor on patents related to the detection, modification, and elimination of senescent cells. CK declares no conflicts of interest. The content is the sole responsibility of the authors and does not necessarily represent the official views of the USDA.
